# The impact of multiple memory formation on dendritic complexity in the hippocampus and anterior cingulate cortex assessed at recent and remote time points

**DOI:** 10.3389/fnbeh.2014.00128

**Published:** 2014-04-21

**Authors:** Brianne C. Wartman, Matthew R. Holahan

**Affiliations:** Department of Neuroscience, Carleton UniversityOttawa, ON, Canada

**Keywords:** hippocampus, anterior cingulate cortex, remote memory, Golgi, memory storage

## Abstract

Consolidation processes, involving synaptic and systems level changes, are suggested to stabilize memories once they are formed. At the synaptic level, dendritic structural changes are associated with long-term memory storage. At the systems level, memory storage dynamics between the hippocampus and anterior cingulate cortex (ACC) may be influenced by the number of sequentially encoded memories. The present experiment utilized Golgi-Cox staining and neuron reconstruction to examine recent and remote structural changes in the hippocampus and ACC following training on three different behavioral procedures. Rats were trained on one hippocampal-dependent task only (a water maze task), two hippocampal-dependent tasks (a water maze task followed by a radial arm maze task), or one hippocampal-dependent and one non-hippocampal-dependent task (a water maze task followed by an operant conditioning task). Rats were euthanized recently or remotely. Brains underwent Golgi-Cox processing and neurons were reconstructed using Neurolucida software (MicroBrightField, Williston, VT, USA). Rats trained on two hippocampal-dependent tasks displayed increased dendritic complexity compared to control rats, in neurons examined in both the ACC and hippocampus at recent and remote time points. Importantly, this behavioral group showed consistent, significant structural differences in the ACC compared to the control group at the recent time point. These findings suggest that taxing the demand placed upon the hippocampus, by training rats on two hippocampal-dependent tasks, engages synaptic and systems consolidation processes in the ACC at an accelerated rate for recent and remote storage of spatial memories.

## Introduction

Consolidation is distinguished into two specific components, synaptic, and systems consolidation. Synaptic consolidation occurs shortly after acquisition and refers to changes at the cellular level (McGaugh, 2000; Kandel, 2001; Dudai, 2004, 2012; Frankland and Bontempi, 2005; Alberini et al., 2006). Systems consolidation refers to a gradual process of reorganization and rearrangement in the brain circuits which support the storage of memories, shifting the storage of memories from an initial encoding circuit to a circuit for long-term storage (McGaugh, 2000; Kandel, 2001; Dudai, 2004, 2012; Frankland and Bontempi, 2005; Alberini et al., 2006).

Synaptic consolidation processes involve the induction of signaling cascades and second messenger systems, protein synthesis and gene expression alterations, all of which may be important synaptic pathways in the initiation of long-term memory storage processes (Kandel, 2001; Dudai, 2004, 2012; Alberini et al., 2006). Once these pathways have been initiated, structural changes, in the form of dendritic complexity, often ensue and are proposed to provide a substrate for the storage of long-term, or remote, memories (Bailey and Kandel, 1993; Trommald et al., 1996; Yang et al., 2009). Therefore, examining differences in dendritic complexity in distinct brain regions following a behavioral task may allow one to infer the involvement of that particular brain region in storing the memory representation for the task.

The hippocampus critically contributes to the initial encoding and storage of memory representations (Scoville and Milner, 1957; Squire, 1992; Cohen and Eichenbaum, 1993). It is hypothesized that as time passes, there is a shift in the balance of neural activity from the hippocampus to cortical regions in the representation of a remote memory. Evidence for the involvement of the anterior cingulate cortex (ACC) in representing remote memories is mounting. Studies have shown increased activity in the ACC on tests for remote (~30 days after encoding) memory (Bontempi et al., 1999; Frankland et al., 2004; Maviel et al., 2004; Teixeira et al., 2006; Lopez et al., 2012; Weible et al., 2012), inactivation of the ACC has been shown to hinder performance on remote memory tests (Bontempi et al., 1999; Frankland et al., 2004; Maviel et al., 2004; Teixeira et al., 2006; Holahan and Routtenberg, 2007; Ding et al., 2008; Restivo et al., 2009; Lopez et al., 2012) and structural changes, indicative of memory storage within the ACC, have been observed at remote time points (Restivo et al., 2009; Vetere et al., 2011; Weible et al., 2012).

The present experiment investigated whether taxing the demand placed upon the hippocampus, by training rats on two hippocampal-dependent tasks, would alter systems consolidation process between the hippocampus and ACC. Rats were trained on one hippocampal-dependent task (the water maze; WM), two different hippocampal-dependent tasks (the WM followed by the radial arm maze; RAM) or one hippocampal-dependent and one non-hippocampal-dependent task (the WM followed by operant conditioning; OP). Because impaired hippocampal function does not alter OP task performance (Shull and Holloway, 1985; Gallagher and Holland, 1992; Corbit and Balleine, 2000; Carvalho et al., 2001) but does impair WM (Morris et al., 1982, 1986; Sutherland et al., 1983, 2001; D'Hooge and De Deyn, 2001; Remondes and Schuman, 2004; Clark et al., 2005a,b; Broadbent et al., 2006; Teixeira et al., 2006; Ramos, 2009; Wiltgen et al., 2010; Holahan and Routtenberg, 2011) and RAM (Jarrard, 1978; Olton et al., 1978; Olton and Papas, 1979; Bouffard and Jarrard, 1986) performance, the OP task is classified as non-hippocampal dependent and the WM and RAM are classified as hippocampal dependent. As such, sequential training on the WM and RAM tasks may result in the activation of similar neural pathways within the hippocampus and result in an accelerated systems consolidation. To examine this hypothesis, dendritic complexity was analyzed in the ACC and CA1 at recent and remote time points using the Golgi-Cox method.

## Materials and methods

### Subjects

Forty-eight Long Evans rats (190–250 g) from Charles River, Quebec were used. Rats were housed individually in clear plastic cages (26 × 20 × 45 cm) and given *ad libitum* water under a 12-h light/dark cycle (lights on at 8:00 a.m.; rats tested during the light phase). Rats received no nesting material and no direct enrichment of any kind in their home cage. Food was restricted until rats reached 90% of their free-feeding baseline, which was maintained throughout the experiment. Prior to behavioral training, rats were given 5 chocolate pellets (45 mg) in their home cage and handled for 5 min daily. Principles of laboratory animal care were followed and all procedures were conducted in accordance with the Canadian Council on Animal Care and protocols approved by the Carleton University Animal Care Committee.

### Apparatus

#### Water maze (WM)

The WM was located in a room within the animal housing area. The opaque, white, polypropylene pool measured 155 cm in diameter and 60 cm in height. The pool was filled to a depth of 37.5 cm with water that remained at approximately 21°C. The “escape” platform was made from clear Plexiglas and submerged approximately 2 cm below the surface of the water. Visual cues such as posters and geometric shapes were located on the walls around the room. The experimenter remained in the same position throughout all trials.

#### Radial arm maze (RAM)

RAM testing was done in a room within the animal housing area, located across the hall from the WM testing room. The maze was positioned 98.5 cm off the floor. Each arm measured 59 cm long and 11 cm wide. The distance between the ends of arms, where food (chocolate pellet, BioServe, New Jersey) reward was located, was 32.5 cm. Plastic inserts were placed on the sides of the maze arms to prevent animals from jumping across arms. Visual cues such as posters and geometric shapes were located on the walls around the room. The experimenter remained in the same position throughout all trials.

#### Operant conditioning (OP)

Rats were tested in groups of six using six operant chambers (Habitest Operant Cage, Coulbourn Instruments; 12”W X 10”D X12”H). Each chamber was housed in an insulated box to minimize external noise. Each chamber possessed a pellet dispensing system, two levers separated by a food hopper, a houselight, a grid floor and a three light-panel. OP training took place in a room outside of the animal housing area.

### Behavioral procedure

**Figure 1:**
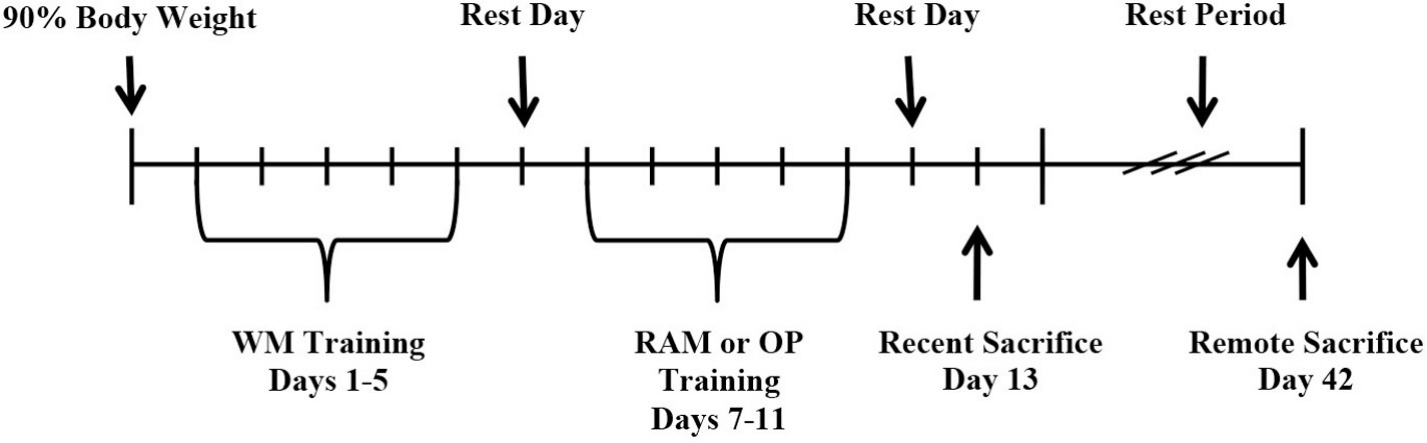
**Timeline of experimental procedures**.

An overview of the behavioral procedure is shown in Figure 1. Rats were assigned to one of three behavioral groups: (1) Training on one hippocampal-dependent task, the WM; (2) Training on two, different, hippocampal-dependent tasks, the WM followed by the RAM; or (3) Training on one hippocampal-dependent task and one non-hippocampal-dependent task, the WM followed by OP. Sequential training on two tasks was separated by a 24 h rest period. A group of control rats received no behavioral training. Following training, rats were sacrificed recently (8 days after the end of WM training, *n* = 24) or remotely (37 days after the end of WM training, *n* = 24). Eight experimental groups resulted (*n* = 6 in each group): (1) Control:Recent (2) WM:Recent (3) WM/RAM:Recent (4) WM/OP:Recent (5) Control:Remote (6) WM:Remote (7) WM/RAM:Remote (8) WM/OP:Remote.

#### Water maze

Rats received 5 training trials per day for 5 days, with a different starting location for every trial within a day and randomized starting locations across days. The hidden platform was located in a fixed location within and across days. Rats were placed in the pool, facing the perimeter, and given a maximum of 60 s to locate the hidden platform. Rats that did not find the hidden platform within 60 s were guided to the platform by the experimenter. All rats remained on the platform for 15 s. Rats then received a 15 s rest period in a holding cage before the next trial. All movement within the pool was tracked using HVS Image 2100 Tracking System (HVS Image, Buckingham, UK). Following the final trial of each day, rats were dried with a towel and placed in a holding cage on a heating pad in the housing room for 10–15 min after which they were returned to the home cage.

#### Radial arm maze

Rats received one day of pretraining and 4 days of testing on the RAM. On the first trial of pretraining, chocolate pellets were located in the starting area, at the entrance to arms, within the arms as well as in the food holes located at the end of each arm. On trial 2 of pretraining, pellets were located within the arms and in food holes only. Trials 3–5 of pretraining had pellets located only in food holes.

Days 2–5 were testing days, where pellets were located in food holes at the ends of 3 of the 5 arms. Baited arms were always the same for an individual rat but differed between rats. Each rat was given 5 trials per day. Trials were a maximum of 5 min each or ended when all food reward had been collected. Rats were placed in a holding cage for 30 s between trials while arms were re-baited. Performance on the maze was manually scored. Sessions were timed, and correct and incorrect arm entries were recorded. An arm entry was defined as all four feet inside an arm.

#### Operant conditioning

Rats in the WM/OP condition were trained to lever-press for chocolate pellets over 5 days, 30 min per day. Upon pressing the lever to the left of the hopper two times (FR2), the house light extinguished, the panel lights above the lever changed from red to green and the pellet dispenser released one 45-mg chocolate pellet (BioServe, New Jersey) into the hopper. Presses on the right lever had no programmed consequences. Presses on the left lever were considered correct and presses on the right lever, incorrect. The number of lever presses, the number of times a rat poked its nose into the hopper and locomotor activity were recorded automatically (Graphic State Notation 2 Version 2.002-00, Coulbourn Instruments).

### Golgi-cox procedure

The Golgi method, originally named the “Black Reaction” was developed by Camillo Golgi in 1873. The invention of the Golgi method marked a huge advancement in the field of neuroscience, largely thanks to Santiago Ramon y Cajal's use of the method. Curiously, and fortunately, the Golgi method stains only a small percentage of cells (estimates range between 1 and 5% Spacek, 1989; Milatovic et al., 2010). This is one of the great advantages of the Golgi method: if every neuron were to become impregnated and stained, it would be impossible to trace a single neuron from its beginning to end amongst all the processes from other neurons. The seemingly random staining seen in the Golgi method provides the opportunity to examine individual neurons. Even presently, it is not well understood why some neurons become impregnated with Golgi solution and why some do not. Some factors suggested to play a role include the functional or metabolic state of a neuron at the time of Golgi fixation or mechanical injury from tissue processing rendering a neuron more susceptible to impregnation (Spacek, 1989; Panese, 2010). The Golgi-Cox method used in this study is a variation on the original Golgi method. This variation uses potassium dichromate and mercuric chloride and is well-suited for analyses in rats (Glaser and Van der Loos, 1981; Gibb and Kolb, 1998). The Golgi-Cox method is considered appropriate for the purposes of studying dendritic morphology (Buell, 1982; Ranjan and Mallick, 2010).

Rats were placed into a Decapicone (Braintree) and decapitated. Brains were rapidly removed and hemisected (hemispheres were counterbalanced). One hemisphere was placed in Golgi-Cox fixative and stored at room temperature, away from light, for 14 days. Following incubation in Golgi-Cox fixative, brains underwent 3 washes in DH_2_O (4 h, 3 h, and 8 h; fresh DH_2_O each time). Next, brains underwent incubation in 10% (8 h), 20% (8 h), and 30% sucrose (minimum 4 days). 200 μm thick sections were sliced on a vibratome and mounted onto gelatinized slides. Slides were placed in a humidified, dark box for 24 h before staining.

Slides were immersed in the following solutions: DH_2_O (1 min), 28% Ammonium Hydroxide (40 min), DH_2_O (1 min), Kodak film fix A (40 min), DH_2_O (2 × 1 min), 50, 70, and 95% ETOH (1 min each), desiccated 100% ETOH (3 × 5 min), desiccated ETOH/Clearene/Chloroform solution (10 min), and desiccated Clearene (2 × 15 min). Slides were coverslipped with generous amounts of Permount mounting medium (Sigma) and placed in a desiccated box to dry for a minimum of 4 days.

### Quantitative neuromorphology

Three pyramidal neurons per rat were analyzed from each brain region. Four rats from each behavioral group were examined. All neuron reconstruction was performed by one experimenter who was unaware of the experimental condition. Brain regions were defined according to Paxinos and Watson (1998). The ACC area of interest was constricted in the anterior–posterior plan from approximately bregma +1.7 to +0.7 mm. The medial border was the midline of the brain and the lateral border was the corpus callosum. Neurons from layer II/III of the ACC were analyzed. The CA1 area of interest focused on the anterior aspect of the dorsal hippocampus and was constricted in the anterior–posterior plan from approximately bregma −3.1 to −4.0 mm.

Neurons to be traced were selected at random, but had to meet predetermined criteria for analysis. Neurons had to be entirely impregnated, staining had to be uniform and complete within processes, the neuron had to be relatively isolated from surrounding impregnated cells or obstructions, and the cell body had to be centrally located within the 200 μm section depth (Anderson et al., 2009). Neurons were reconstructed at 100X magnification using Neurolucida software (MicroBrightField, Williston, VT, USA). Tracings began at the cell body. Apical and basal dendrites were traced in their entirety and all visible spines were marked. For each neuron, dendritic length, total number of branches, and total number of spines were analyzed separately in apical and basal dendrites.

### Statistical analyses

Four rats from each behavioral group were examined; three neurons per rat per brain area (ACC and CA1) were analyzed. Thus, resulting in 12 neurons per behavioral condition per brain region (i.e., 96 reconstructed neurons from the ACC and 96 reconstructed neurons from the CA1). For statistical analysis, the measurements of the three analyzed neurons per rat per brain region were pooled to get one value for each rat (*n* = 4 per behavioral group). Separate analyses were carried out in the ACC and CA1 of the hippocampus and apical and basal dendrites were analyzed separately. Two-Way ANOVAs in each region for apical and basal dendrites were run with Behavioral group (Control, WM Only, WM/RAM, or WM/OP) and Time (Recent or Remote) as the fixed factors and the characteristic of interest (dendritic length, total number of branches, and total number of spines) as the dependent variables.

## Results

### Behavioral training

**Figure 2:**
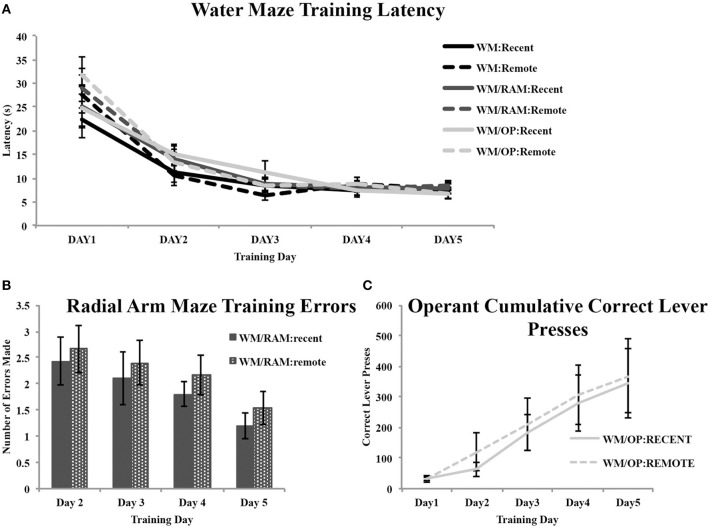
**Behavioral training data. (A)** All rats received water maze training. Average latencies to reach the hidden platform during the 5 days of water maze training are displayed. A main effect of day was found. Latency to reach the hidden platform decreased across training day indicating an improvement in performance. No main effect of behavior or time was found. **(B)** Average number of errors (unbaited arm entries and re-entries) made in the radial arm maze task during the 4 days of training. A main effect of day was found. Errors made decreased across training day indicating an improvement in performance. No main effect of time was found. **(C)** Average correct lever presses on the operant conditioning task during the 5 days of training. A main effect of day was found. Correct lever presses increased across training day indicating an improvement in performance. No main effect of time was found.

Figure 2 shows acquisition data for the WM, RAM, and OP tasks. All rats received WM training. Latency to reach the hidden platform was recorded (Figure 2A). A repeated measures ANOVA with day (1–5) as the within-subject factor and behavior (WM Only, WM/RAM, or WM/OP) and time (recent or remote) as the between-subject factors revealed a main effect of day [*F*_(4, 120)_ = 112.88, *p* < 0.001] but no main effect of behavior or time.

Two groups received training on the WM followed by training on the RAM: one assigned to receive a recent WM probe test and one assigned to receive a remote WM probe (Figure 2B). The number of errors (unbaited arm entries and re-entries) made during training was analyzed with a repeated measures ANOVA with day (2–4) as the within-subject factor and time (recent or remote) as the between-subject factor. Analyses revealed a main effect of day [*F*_(3, 30)_ = 2.82, *p* = 0.05] but no main effect of time.

Two groups received training on the WM followed by training on the operant task: one assigned to receive a recent WM probe test and one assigned to receive a remote WM probe (Figure 2C). The number of correct lever presses made during training was analyzed with a repeated measures ANOVA with day (1–5) as the within-subject factor and time (recent or remote) as the between-subject factor. Analyses revealed a main effect of day [*F*_(4, 40)_ = 12.73, *p* < 0.001] but no main effect of time.

### Neuron morphology

#### Cell body size

Cell body size in the ACC was analyzed using a Two-Way ANOVA with Behavior (Control, WM Only, WM/RAM or WM/OP) and time (recent or remote) as the independent variables and cell body size as the dependent variable. No main effect of behavior or time was found (data not shown).

Cell body size in the CA1 of the hippocampus was analyzed using a Two-Way ANOVA with Behavior (Control, WM Only, WM/RAM, or WM/OP) and time (recent or remote) as the independent variables and cell body size as the dependent variable. No main effect of behavior or time was found (data not shown).

#### Anterior cingulate cortex

**Figure 3:**
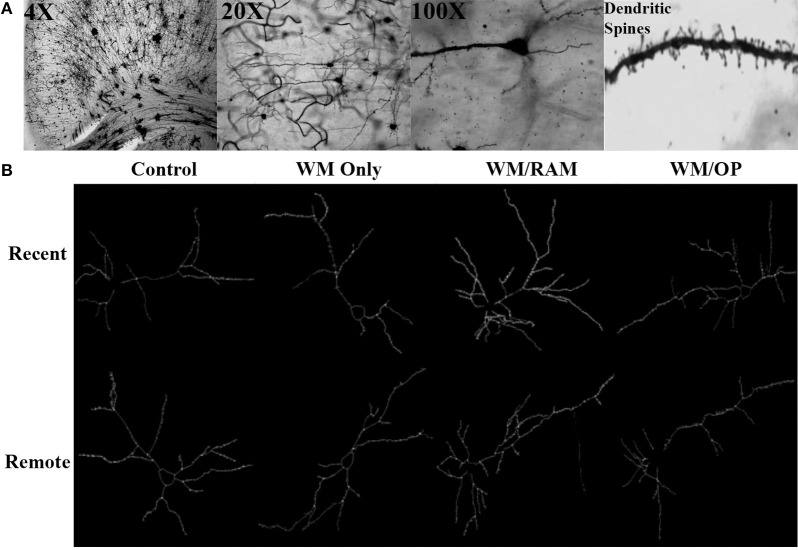
**(A)** Images of 4X, 20X, and 100X magnified Golgi impregnated neurons in the ACC. A zoomed-in image of an apical dendrite with dendritic spines is shown in the last panel. **(B)** Representative graphics of Golgi impregnated neurons in the ACC, traced using Neurolucida software.

Figure 3 displays images of Golgi impregnated neurons and representative graphics of Golgi impregnated neurons from each of the behavioral groups in the ACC.

**Figure 4:**
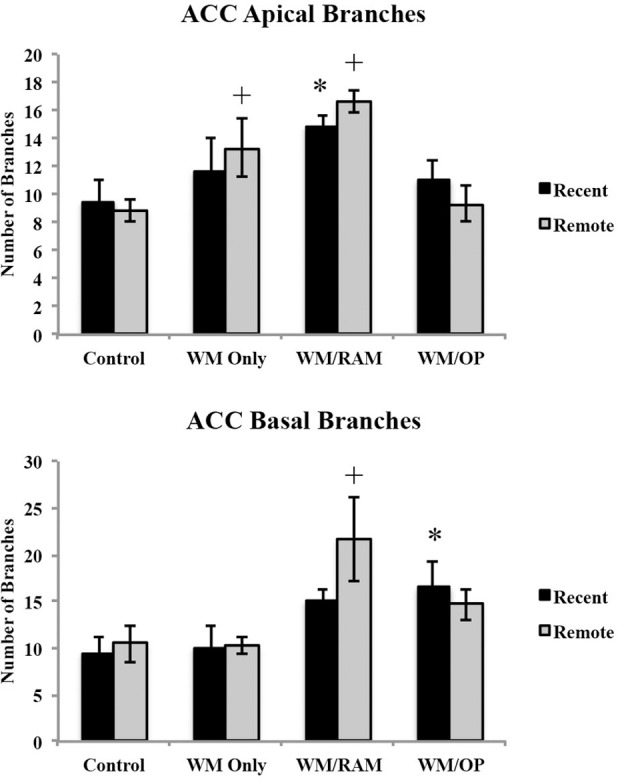
**Total number of branches in the ACC in apical and basal dendrites**. Only significant differences between behavioral groups and their respective control group are displayed. Refer to Results section for all comparison results. ^*^Significantly different from Control:Recent group (*p* < 0.05); ^+^Significantly different from Control:Remote (*p* < 0.05).

***Number of branches.*** Figure 4 displays total number of branches in the ACC in apical and basal dendrites.

***Apical.*** A Two-Way ANOVA with Behavioral group (Control, WM Only, WM/RAM, or WM/OP) and time (Recent or Remote) as the fixed factors and number of branches as the dependent variable revealed a main effect of behavior [*F*_(3, 24)_ = 7.46, *p* < 0.001] with group WM/RAM displaying the greatest number of branches (16). Fisher's LSD *post-hoc* analyses revealed group WM/RAM:Recent had significantly greater number of branches compared to groups WM/OP:Remote, Control:Recent, and Control:Remote (*p* < 0.05). Group WM/RAM:Remote had significantly greater number of branches compared to groups WM Only:Recent, WM/OP:Recent, WM/OP:Remote, Control:Recent, and Control:Remote (*p* < 0.05). Group WM Only:Remote had significantly greater number of branches compared to group Control:Remote (*p* < 0.05).

***Basal.*** A Two-Way ANOVA with Behavioral group (Control, WM Only, WM/RAM, or WM/OP) and time (Recent or Remote) as the fixed factors and number of branches as the dependent variable revealed a main effect of behavior [*F*_(3, 24)_ = 5.98, *p* < 0.01], with group WM/RAM displaying the greatest number of branches (18). Fisher's LSD *post-hoc* analyses revealed group WM/RAM:Remote had significantly greater number of branches compared to groups from WM Only:Recent, WM Only:Remote, WM/OP:Remote, Control:Recent, and Control:Remote, (*p* = 0.05). Group WM/OP:Recent had significantly greater number of branches compared to group Control:Recent (*p* < 0.05).

**Figure 5:**
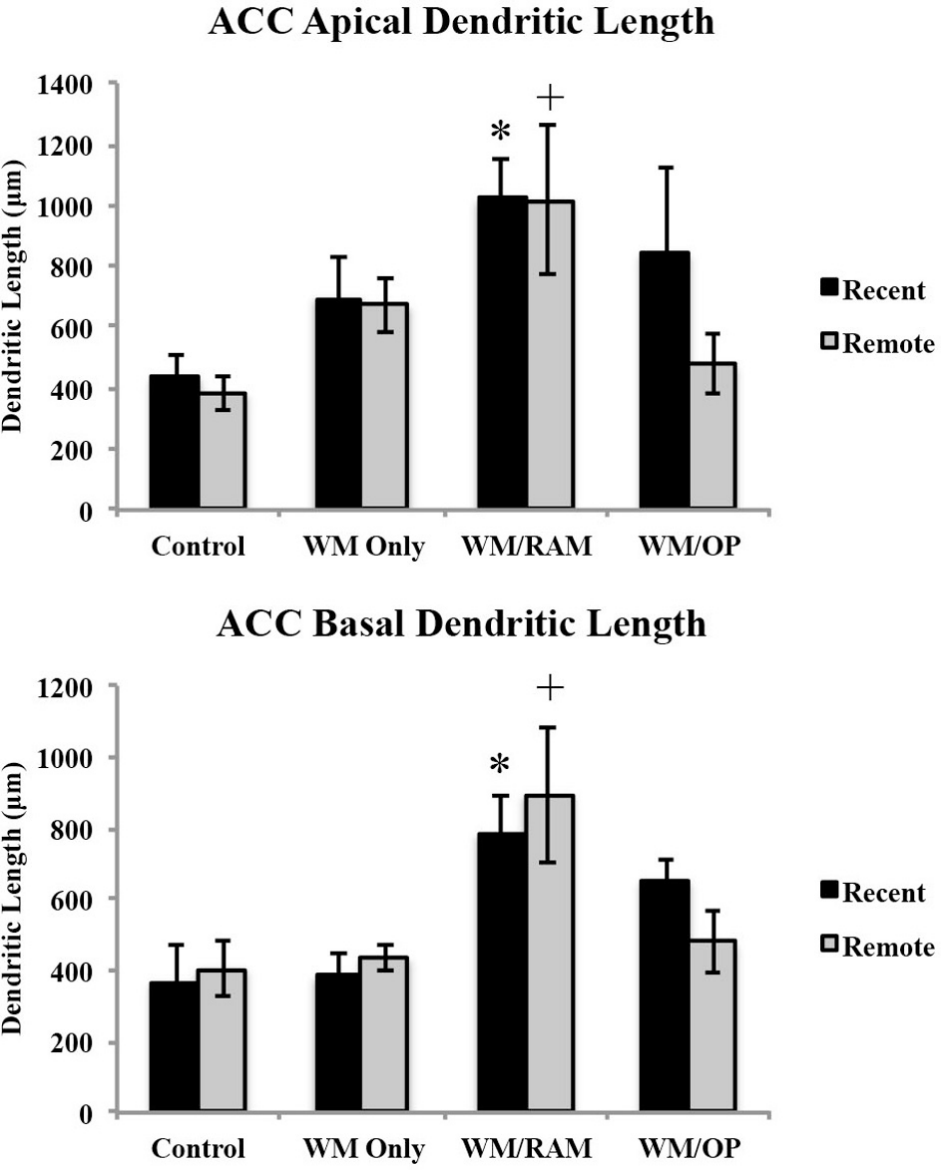
**Total dendritic length in the ACC in apical and basal dendrites**. Only significant differences between behavioral groups and their respective control group are displayed. Refer to Results section for all comparison results. ^*^Significantly different from Control:recent group (*p* < 0.05); ^+^Significantly different from Control:Remote (*p* < 0.05).

***Dendritic Length.*** Figure 5 displays total dendritic length in the ACC in apical and basal dendrites.

***Apical.*** A Two-Way ANOVA with Behavioral group (Control, WM Only, WM/RAM, or WM/OP) and time (Recent or Remote) as the fixed factors and dendritic length as the dependent variable revealed a main effect of behavior [*F*_(3, 24)_ = 5.00, *p* < 0.01], with group WM/RAM displaying the greatest dendritic length (1016 μm). Fisher's LSD *post-hoc* analyses revealed group WM/RAM:Recent had significantly greater dendritic length compared to groups WM/OP:Remote, Control:Recent, and Control:Remote (*p* < 0.05). Group WM/RAM:Remote had significantly greater dendritic length compared to groups WM/OP:Remote, Control:Recent, Control:Remote (*p* < 0.05). Group WM/OP:Recent had significantly greater dendritic length compared to group Control:Remote (*p* = 0.05).

***Basal.*** A Two-Way ANOVA with Behavioral group (Control, WM Only, WM/RAM, or WM/OP) and time (Recent or Remote) as the fixed factors and dendritic length as the dependent variable revealed a main effect of behavior [*F*_(3, 24)_ = 8.52, *p* < 0.001], with group WM/RAM displaying the greatest dendritic length (839 μm). Fisher's LSD *post-hoc* analyses revealed group WM/RAM:Recent had significantly greater dendritic length compared to groups WM Only:Recent, WM Only:Remote, WM/OP:Remote, Control:Recent, and Control:Remote (*p* < 0.05). Group WM/RAM:Remote had significantly greater dendritic length compared to groups WM Only:Recent, WM Only:Remote, WM/OP:Remote, Control:Recent and Control:Remote (*p* < 0.05).

**Figure 6:**
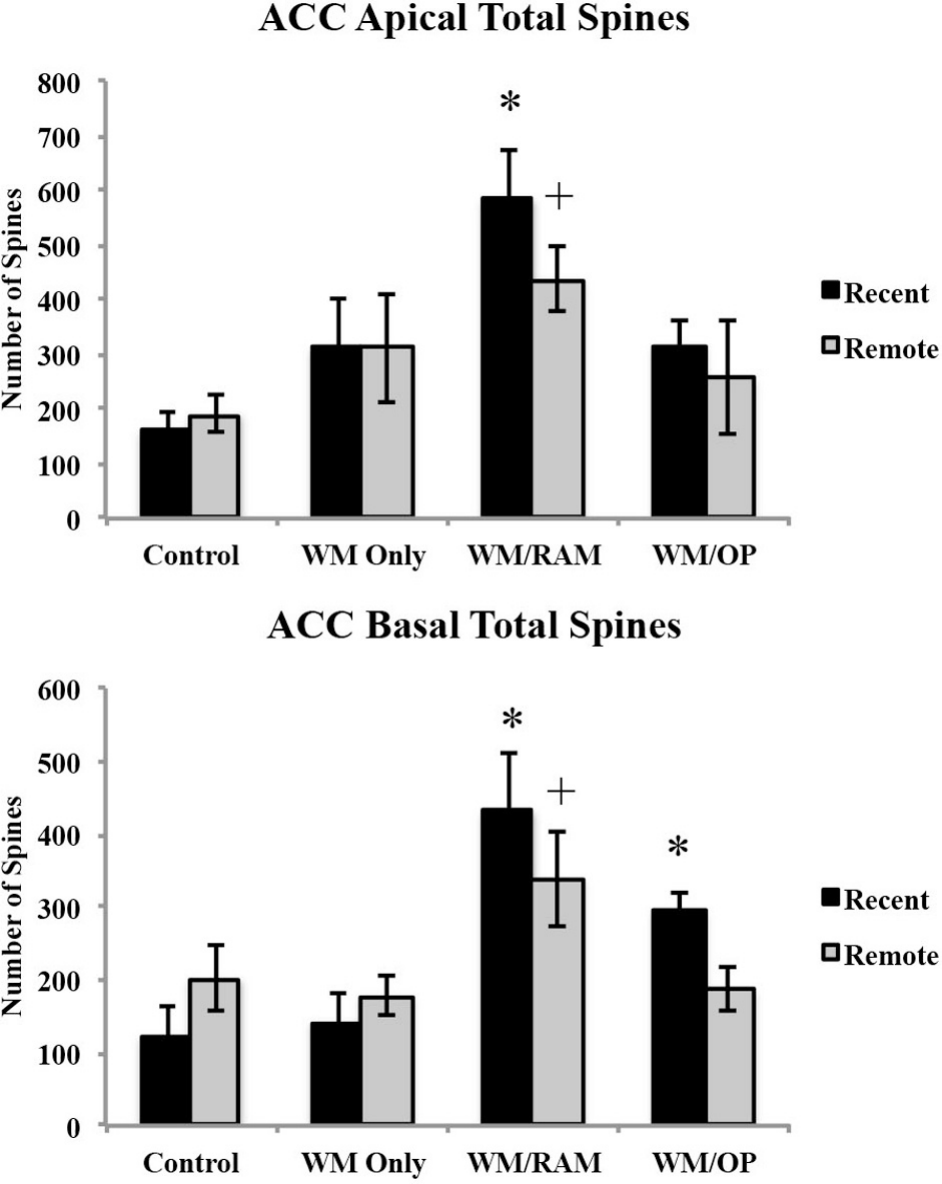
**Total number of spines in the ACC in apical and basal dendrites**. Only significant differences between behavioral groups and their respective control group are displayed. Refer to Results section for all comparison results. ^*^Significantly different from Control:recent group (*p* < 0.05); ^+^Significantly different from Control:Remote (*p* < 0.05).

***Number of spines.*** Figure 6 displays total number of spines in the ACC in apical and basal dendrites.

***Apical.*** A Two-Way ANOVA with Behavioral group (Control, WM Only, WM/RAM, or WM/OP) and time (Recent or Remote) as the fixed factors and number of spines as the dependent variable revealed a main effect of behavior [*F*_(3, 24)_ = 6.92, *p* < 0.01], with group WM/RAM displaying the greatest number of spines (511). Fisher's LSD *post-hoc* analyses revealed group WM/RAM:Recent had significantly greater number of spines compared to groups WM Only:Recent, WM Only:Remote, WM/OP:Recent, WM/OP:Remote, Control:Recent and Control:Remote (*p* < 0.05). Group WM/RAM:Remote had significantly greater number of spines compared to groups Control:Recent and Control:Remote (*p* < 0.05).

***Basal.*** A Two-Way ANOVA with Behavioral group (Control, WM Only, WM/RAM, or WM/OP) and time (Recent or Remote) as the fixed factors and number of spines as the dependent variable revealed a main effect of behavior [*F*_(3, 24)_ = 10.23, *p* < 0.001], with group WM/RAM displaying the greatest number of spines (385). Fisher's LSD *post-hoc* analyses revealed group WM/RAM:Recent had significantly greater number of spines compared to groups WM Only:Recent, WM Only:Remote, WM/OP:Recent, WM/OP:Remote, Control:Recent, Control:Remote (*p* < 0.05). Group WM/RAM:Remote had significantly greater number of spines compared to groups WM Only:Recent, WM Only:Remote, Control:Recent, and Control:Remote (*p* < 0.05). Group WM/OP:Recent had significantly greater number of spines compared to groups WM Only:Recent and Control:Recent (*p* < 0.05).

**Figure 7:**
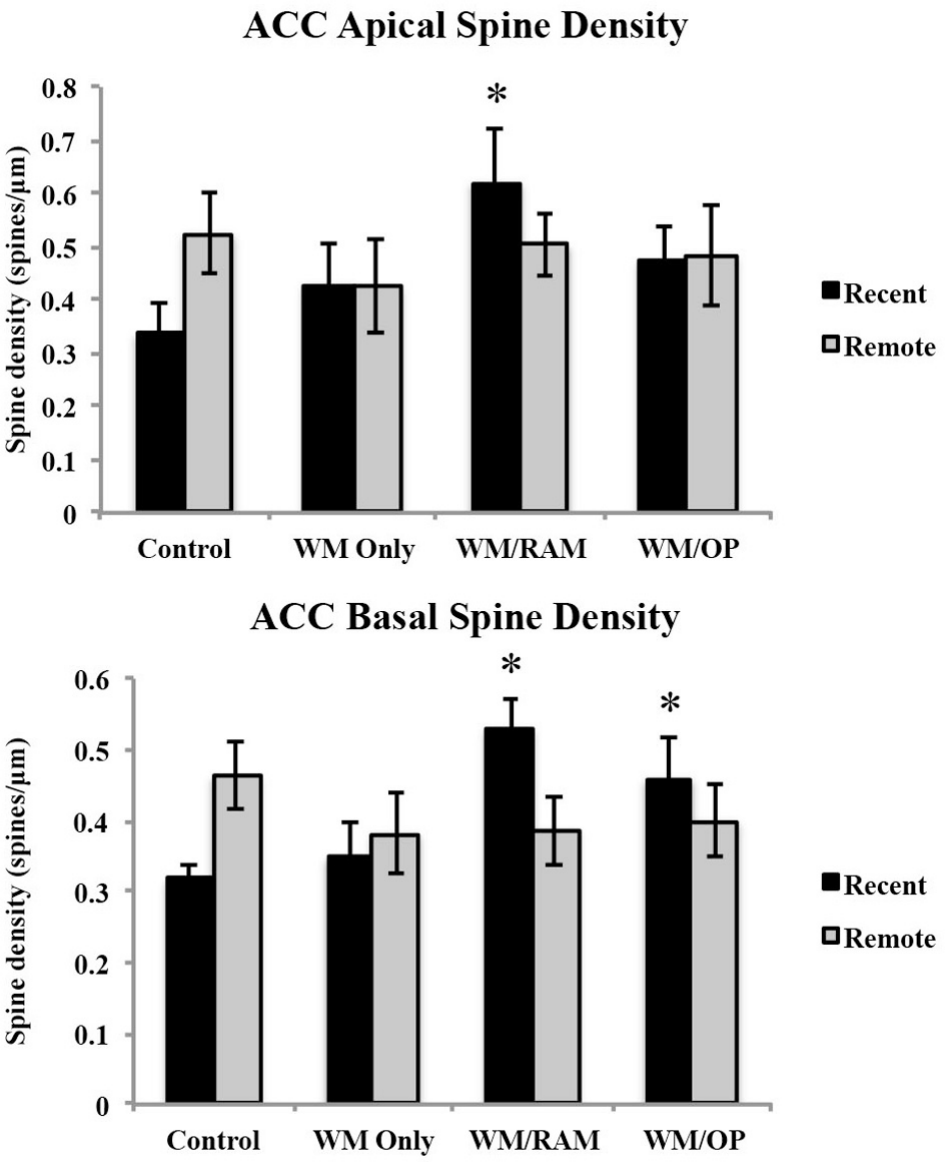
**Spine density in the ACC in apical and basal dendrites**. Only significant differences between behavioral groups and their respective control group are displayed. Refer to Results section for all comparison results. ^*^Significantly different from Control:recent group (*p* < 0.05).

***Spine density.*** Figure 7 displays spine density in the ACC in apical and basal dendrites.

***Apical.*** A Two-Way ANOVA with Behavioral group (Control, WM Only, WM/RAM, or WM/OP) and time (Recent or Remote) as the fixed factors and spine density as the dependent variable revealed no main effects. Planned Fisher's LSD *post-hoc* comparisons revealed group WM/RAM:Recent had significantly greater spine density compared to group Control:Recent (*p* < 0.05).

***Basal.*** A Two-Way ANOVA with Behavioral group (Control, WM Only, WM/RAM or WM/OP) and time (Recent or Remote) as the fixed factors and spine density as the dependent variable revealed no main effects. A significant interaction between behavior X time [*F*_(3, 24)_ = 3.39, *p* < 0.05] was found. Fisher's LSD *post-hoc* comparisons revealed group WM/RAM:Recent had significantly greater spine density compared to groups WM Only:Recent, WM Only:Remote, WM/RAM:Remote, and Control:Recent (*p* < 0.05). Group WM/OP:Recent had significantly greater spine density compared to group Control:Recent (*p* < 0.05). Group Control:Remote had significantly greater spine density compared to group Control:Recent (*p* < 0.05).

***Summary of ACC findings.*** Group WM/RAM displayed the greatest and most consistent dendritic complexity in the ACC. At the recent time point, group WM/RAM was the sole group to show significantly increased dendritic complexity in apical dendrites compared to the control group.

#### CA1 of the hippocampus

**Figure 8:**
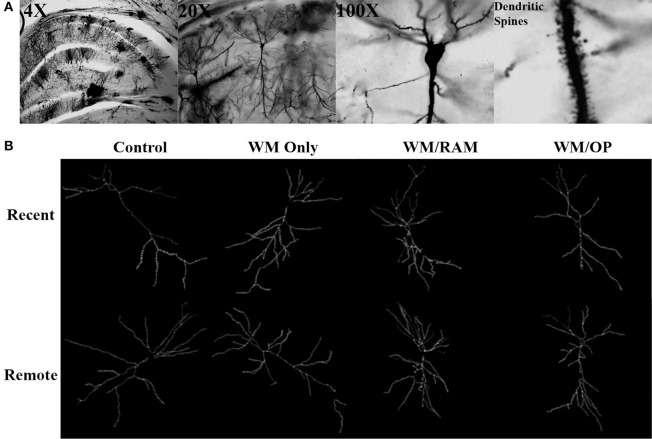
**(A)** Images of 4X, 20X, and 100X magnified Golgi impregnated neurons in the CA1. A zoomed-in image of an apical dendrite with dendritic spines is shown in the last panel. **(B)** Representative graphics of Golgi impregnated neurons in the CA1, traced using Neurolucida software.

Figure 8 displays images of Golgi impregnated neurons and representative graphics of Golgi impregnated neurons from each of the behavioral groups in the CA1.

**Figure 9:**
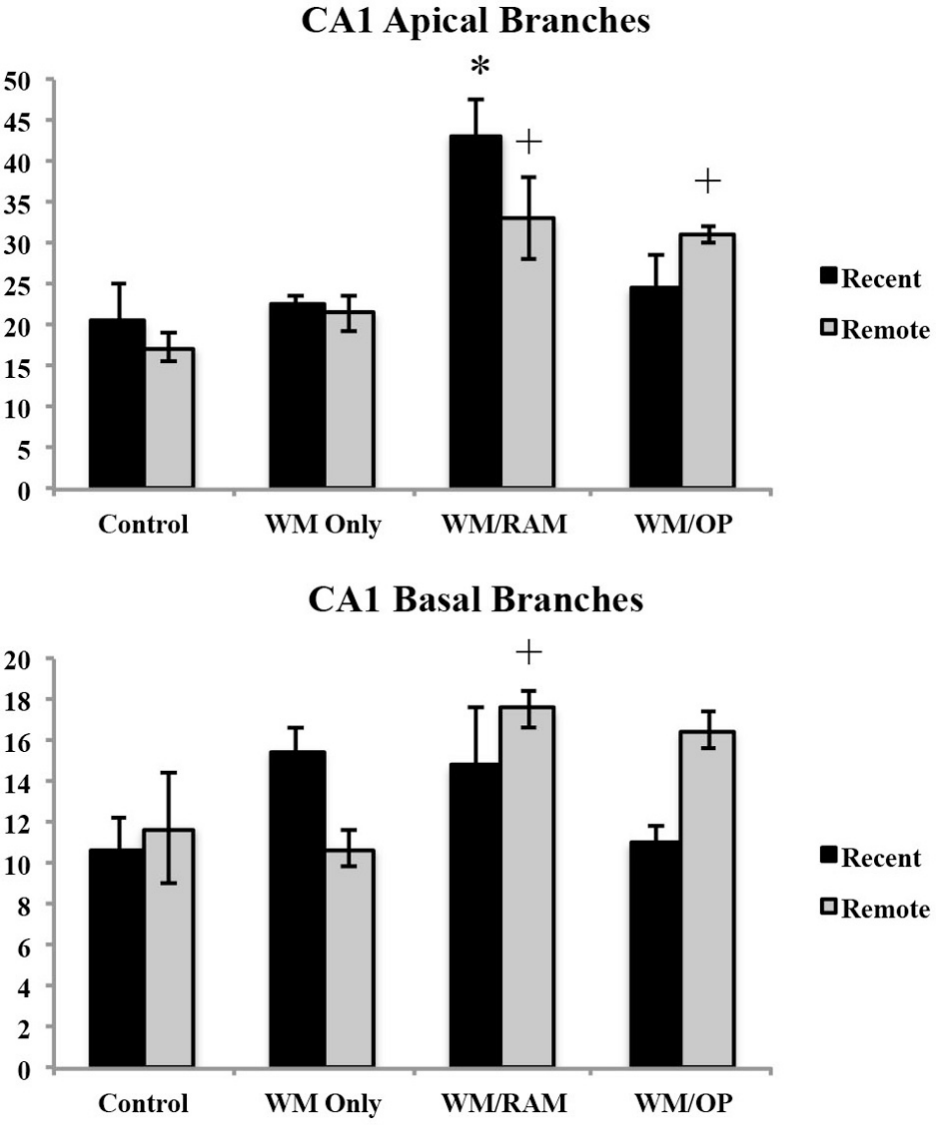
**Total number of branches in the CA1 in apical and basal dendrites**. Only significant differences between behavioral groups and their respective control group are displayed. Refer to Results section for all comparison results. ^*^Significantly different from Control:recent group (*p* < 0.05); ^+^Significantly different from Control:Remote (*p* < 0.05).

***Number of branches.*** Figure 9 displays total number of branches in the CA1 in apical and basal dendrites.

***Apical.*** A Two-Way ANOVA with Behavioral group (Control, WM Only, WM/RAM, or WM/OP) and time (Recent or Remote) as the fixed factors and number of branches as the dependent variable revealed a main effect of behavior [*F*_(3, 24)_ = 12.63, *p* < 0.001] with group WM/RAM displaying the greatest number of branches (38). Fisher's LSD *post-hoc* analyses revealed group WM/RAM:Recent had significantly greater number of branches compared to every other behavioral group (*p* < 0.05). Group WM/RAM:Remote had significantly greater number of branches compared to groups WM Only:Recent, WM Only:Remote, WM/RAM:Recent, Control:Recent, and Control:Remote (*p* < 0.05). Group WM/OP:Recent had significantly greater number of branches compared to group WM/OP:Remote (*p* < 0.05). Group WM/OP:Remote had significantly greater number of branches compared to groups WM/RAM:Recent, Control:Recent, and Control:Remote (*p* < 0.05).

***Basal.*** A Two-Way ANOVA with Behavioral group (Control, WM Only, WM/RAM, or WM/OP) and time (Recent or Remote) as the fixed factors and number of branches as the dependent variable revealed a main effect of behavior [*F*_(3, 24)_ = 2.98, *p* = 0.05] with group WM/RAM displaying the greatest number of branches (16). A significant interaction between behavior X time was found [*F*_(3, 24)_ = 3.42, *p* < 0.05]. Fisher's LSD *post-hoc* analyses revealed group WM/RAM:Remote had significantly greater number of branches compared to groups WM Only:Remote, WM/OP:Recent, Control:Recent, and Control:Remote (*p* < 0.05). Group WM/OP:Recent had significantly fewer number of branches compared to groups WM/RAM:Remote, WM/OP:Remote (*p* < 0.05). Group WM/OP:Remote had significantly greater number of branches compared to group WM Only:Remote, WM/OP:Recent, and Control:Recent (*p* < 0.05).

**Figure 10:**
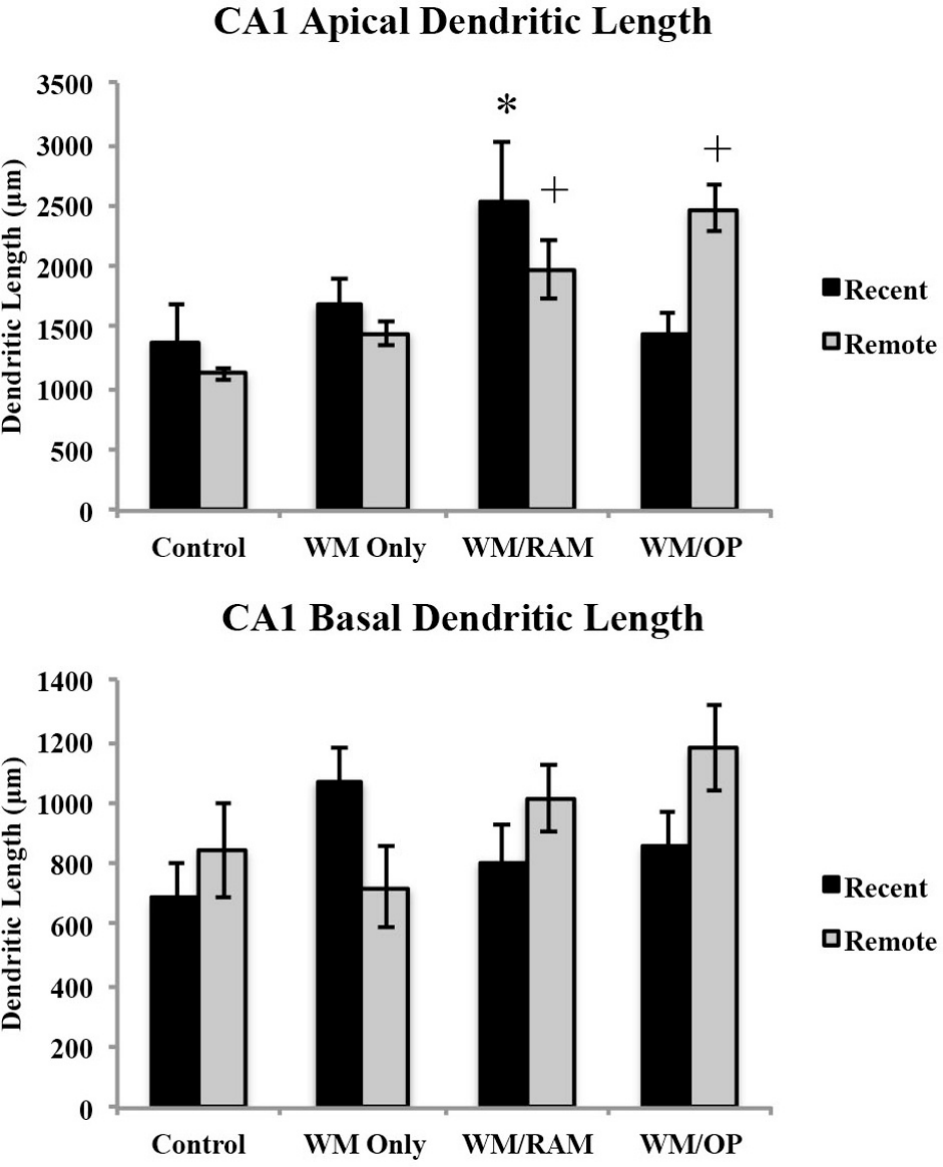
**Total dendritic length in the CA1 in apical and basal dendrites**. Only significant differences between behavioral groups and their respective control group are displayed. Refer to Results section for all comparison results. ^*^Significantly different from Control:recent group (*p* < 0.05); ^+^Significantly different from Control:Remote (*p* < 0.05).

***Dendritic length.*** Figure 10 displays total dendritic length in the CA1 in apical and basal dendrites.

***Apical.*** A Two-Way ANOVA with Behavioral group (Control, WM Only, WM/RAM, or WM/OP) and time (Recent or Remote) as the fixed factors and dendritic length as the dependent variable revealed a main effect of behavior [*F*_(3, 24)_ = 6.24, *p* < 0.01], with group WM/RAM displaying the greatest dendritic length (2259 μm). A significant interaction between behavior X time was found [*F*_(3, 24)_ = 4.04, *p* < 0.05]. Fisher's LSD *post-hoc* analyses revealed group WM/RAM:Recent had significantly greater dendritic length compared to groups WM Only:Recent, WM Only:Remote, WM/OP:Recent, Control:Recent, and Control:Remote (*p* < 0.05). Group WM/RAM:Remote had significantly greater dendritic length compared to group Contol:Remote (*p* < 0.05). Group WM/OP:Remote had significantly greater dendritic length compared to groups WM Only:Recent, WM Only:Remote, WM/OP:Recent, Control:Recent, and Control:Remote (*p* < 0.05).

***Basal.*** A Two-Way ANOVA with Behavioral group (Control, WM Only, WM/RAM, or WM/OP) and time (Recent or Remote) as the fixed factors and dendritic length as the dependent variable revealed no main effects. Planned Fisher's LSD *post-hoc* comparisons revealed group WM/OP:Remote had significantly greater dendritic length compared to groups WM Only:Remote and Control:Recent. Group Control:Recent had significantly less dendritic length compared to groups WM Only:Recent and WM/OP:Remote (*p* < 0.05).

**Figure 11:**
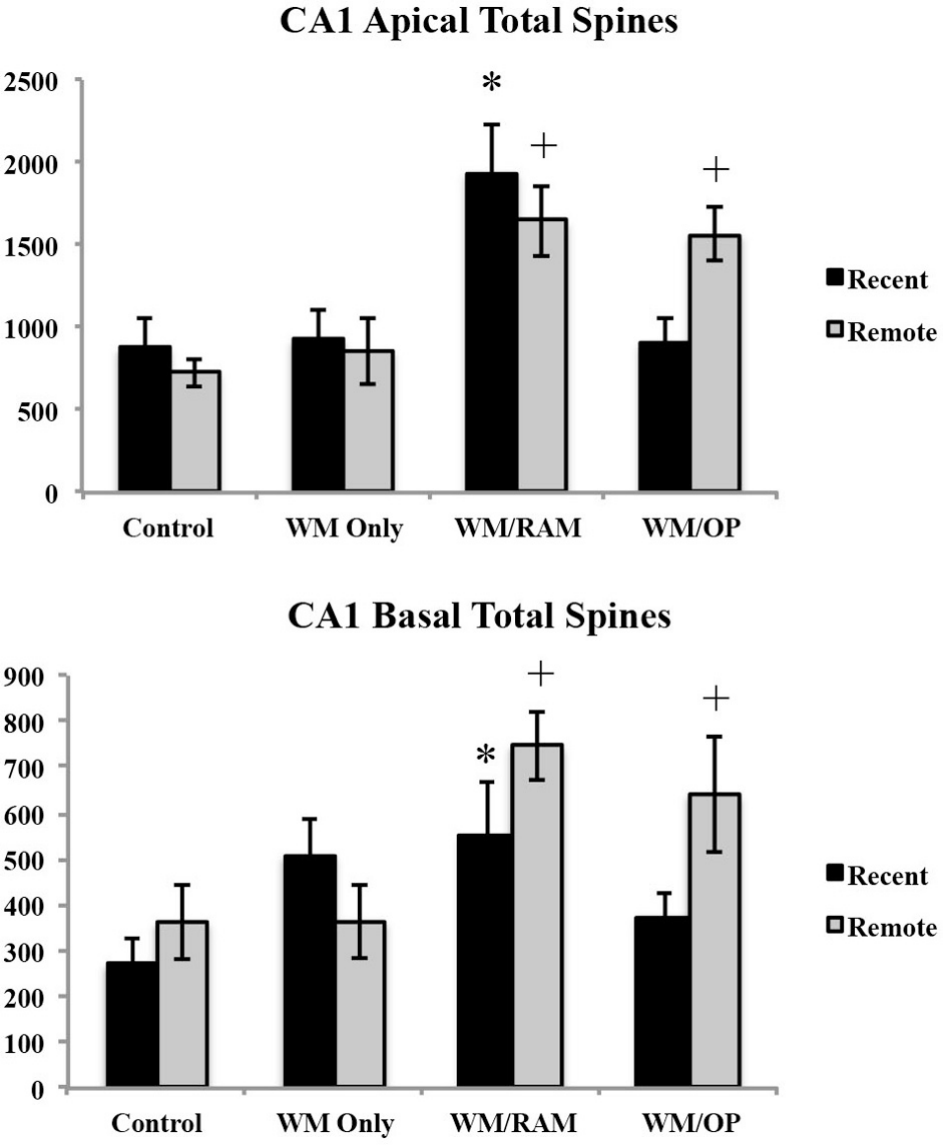
**Total number of spines in the ACC in apical and basal dendrites**. Only significant differences between behavioral groups and their respective control group are displayed. Refer to Results section for all comparison results. ^*^Significantly different from Control:recent group (*p* < 0.05); ^+^Significantly different from Control:Remote (*p* < 0.05).

***Number of spines.*** Figure 11 displays total number of spines in the CA1 in apical and basal dendrites.

***Apical.*** A Two-Way ANOVA with Behavioral group (Control, WM Only, WM/RAM, or WM/OP) and time (Recent or Remote) as the fixed factors and number of spines as the dependent variable revealed a main effect of behavior [*F*_(3, 24)_ = 10.81, *p* < 0.001], with group WM/RAM displaying the greatest number of spines (1793). Fisher's LSD *post-hoc* analyses revealed group WM/RAM:Recent had significantly greater number of spines compared to groups WM Only:Recent, WM Only:Remote, WM/OP:Recent, Control:Recent, and Control:Remote (*p* < 0.05). Group WM/RAM:Remote had significantly greater number of spines compared to group WM Only:Recent, WM Only:Remote, WM/OP:Reent, Control:Recent, and Control:Remote (*p* < 0.05). Group WM/OP:Recent had significantly fewer number of spines compared to groups WM/RAM:Recent, WM/RAM:Remote and WM/OP:Remote (*p* < 0.05). Group WM/OP:Remote had significantly greater number of spines compared to groups WM Only:Recent, WM Only:Remote, WM/OP:Recent, Control:Recent, and Control:Remote (*p* < 0.05).

***Basal.*** A Two-Way ANOVA with Behavioral group (Control, WM Only, WM/RAM, or WM/OP) and time (Recent or Remote) as the fixed factors and number of spines as the dependent variable revealed a main effect of behavior [*F*_(3, 24)_ = 5.07, *p* < 0.01], with group WM/RAM displaying the greatest number of spines (647). Fisher's LSD *post-hoc* analyses revealed group WM/RAM:Recent had significantly greater number of spines compared to group Control:Recent (*p* < 0.05). Group WM/RAM:Remote had significantly greater number of spines compared to groups WM Only:Remote, WM/OP:Recent, Control:Recent, and Control:Remote (*p* < 0.05). Group WM/OP:Recent had significantly fewer number of spines compared to groups WM/RAM:Remote and WM/OP:Remote (*p* < 0.05). Group WM/OP:Remote had significantly greater number of spines compared to groups WM Only:Remote, WM/OP:Recent, Control:Recent, and Control:Remote (*p* < 0.05).

**Figure 12:**
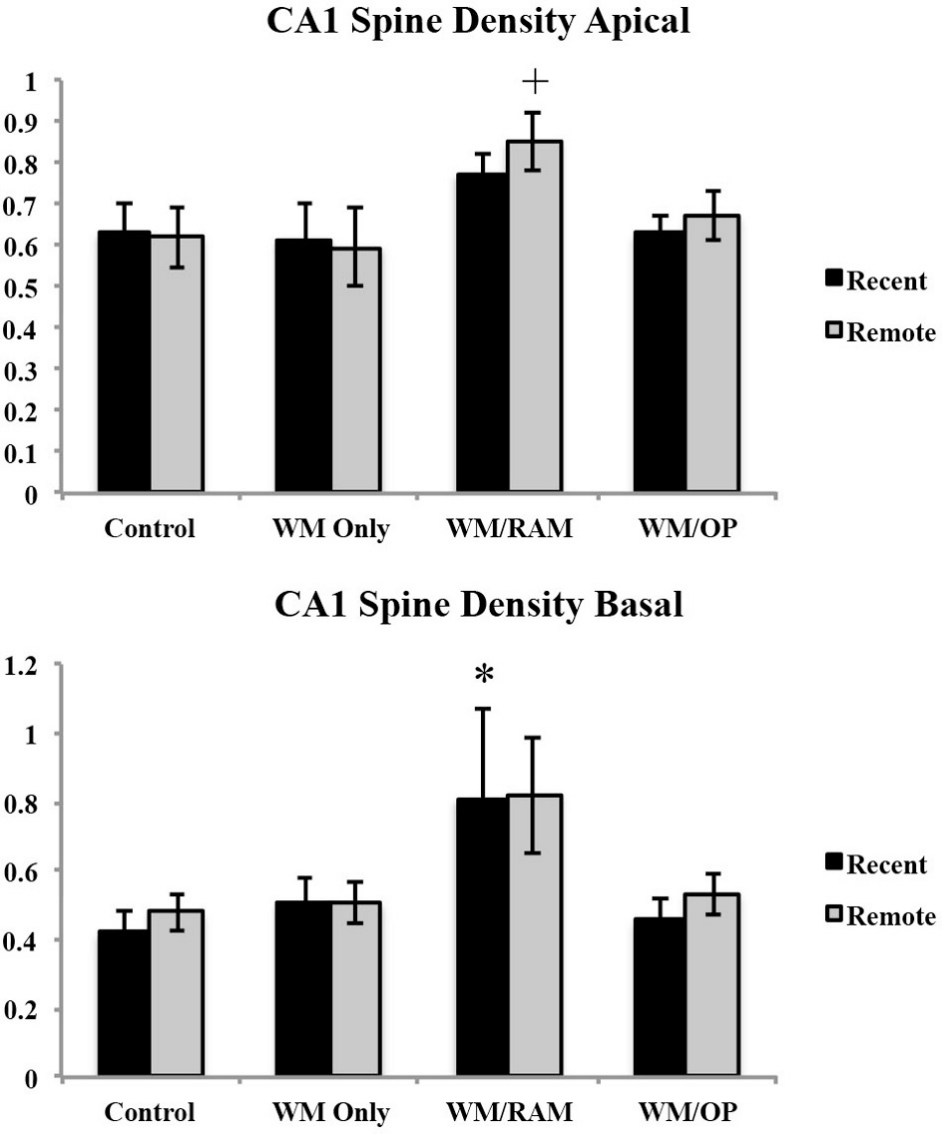
**Spine density in the CA1 in apical and basal dendrites**. Only significant differences between behavioral groups and their respective control group are displayed. Refer to Results section for all comparison results. ^*^Significantly different from Control:recent group (*p* < 0.05); ^+^Significantly different from Control:Remote (*p* < 0.05).

***Spine density.*** Figure 12 displays spine density in the CA1 in apical and basal dendrites.

***Apical.*** A Two-Way ANOVA with Behavioral group (Control, WM Only, WM/RAM or WM/OP) and time (Recent or Remote) as the fixed factors and spine density as the dependent variable revealed a main effect of behavior [*F*_(3, 24)_ = 3.42, *p* < 0.05], with group WM/RAM displaying the greatest spine density (0.812). Fisher's LSD *post-hoc* analyses revealed group WM/RAM:Remote had significantly greater spine density compared to groups WM Only:Recent, WM Only:Remote, WM/OP:Recent, Control:Recent, and Control:Remote (*p* < 0.05).

***Basal.*** A Two-Way ANOVA with Behavioral group (Control, WM Only, WM/RAM, or WM/OP) and time (Recent or Remote) as the fixed factors and spine density as the dependent variable revealed a main effect of behavior [*F*_(3, 24)_ = 3.63, *p* < 0.05], with group WM/RAM displaying the greatest spine density (0.812). Fisher's LSD *post-hoc* analyses revealed group WM/RAM:Recent had significantly greater spine density compared to group Control:Recent (*p* < 0.05). Group WM/RAM:Remote had significantly greater spine density compared to groups WM/OP:Recent and Control:Recent (*p* < 0.05).

***Summary of CA1 findings.*** Group WM/RAM displayed the greatest dendritic complexity in the CA1. Group WM/RAM was the only group to show increased dendritic complexity at the recent time point. Group WM/RAM also showed persistent increases in the dendritic complexity characteristics analyzed as this group was found to have these characteristics significantly increased from the Control group at the remote time point as well. Group WM/OP:Remote consistently showed increases in dendritic complexity at the remote time point.

## Discussion

A great deal of human research supports the idea of a time-limited role for the hippocampus in memory storage (Scoville and Milner, 1957; Kapur and Brooks, 1999; Teng and Squire, 1999; Rosenbaum et al., 2000; Manns et al., 2003; Bayley et al., 2006). Within animal research, some evidence suggests hippocampal damage results in temporally graded retrograde amnesia (Kim and Fanselow, 1992; Frankland et al., 2004; Maviel et al., 2004; Teixeira et al., 2006; Ding et al., 2008) whereas other evidence suggests hippocampal damage results in a flat, or ungraded, retrograde amnesia (Mumby et al., 1999; Sutherland et al., 2001, 2008; Clark et al., 2005a,b, 2007; Broadbent et al., 2006; Lehmann et al., 2007, 2013; Epp et al., 2008; Sparks et al., 2011). We propose that the number of memories processed over the lifetime underlies this discrepancy. In humans, there is a seemingly endless sequence of memory storage and retrieval processes, while laboratory rats and mice are typically required to retrieve and store only one memory for one behavioral task. In the absence of multiple memories competing for processing “space” in the hippocampus, a hippocampal-dependent memory may not be as likely to require, and thus form, extra-hippocampal (cortical) connections, thereby relying heavily on hippocampal involvement for an extended period of time. Processing of several hippocampal-dependent memories (resulting from multiple hippocampal-dependent tasks) may compete for hippocampal processing space, resulting in an increased reliance on cortical structures, such as the ACC.

Dendrites represent structures that serve to receive and integrate information (Jan and Jan, 2001). As such, structural changes in dendrites may partially fulfill the neural requirements necessary to maintain memory representations for the long term (Bailey and Kandel, 1993; Trommald et al., 1996). Changes in the dendritic characteristics examined (dendritic length, branching, number of spines, and spine density) collectively grouped as dendritic complexity, serve to increase the available surface area for synaptic contact, increasing the capacity for neuronal connectivity (Ramón y Cajal, 1911). Greater dendritic length and greater dendritic branching increase the number of possible synaptic contacts and information that may be received by a dendrite (Jan and Jan, 2001). Alterations in dendritic characteristics may reflect increased or decreased responses to incoming synaptic information.

Spine remodeling, which includes both spine formation and elimination, has been suggested to play an important role in structural plasticity (Yang et al., 2009). Yang et al. (2009) demonstrated that only a small proportion of new spines that result from novel experiences persist and survive. Thus, number of spines or spine density may not always be an effective indicator of memory storage or retention on its own; spine formation may represent a transient event following experience. Likely, the type of spine (thin, mushroom, etc.) is of greater importance for memory persistence. Unfortunately, spine type was not included in the analysis in the present study as technical limitations prohibited this type of examination.

## Results

Examination of neuron morphology included total dendritic length, number of branches, number of spines, and spine density (Hamilton et al., 2010) in the ACC and CA1. Overall, group WM/RAM displayed the most robust increases on all indices evaluated compared to the control groups in both the ACC and CA1. This suggests a greater degree of dendritic complexity in this group. Importantly, group WM/RAM consistently showed increased dendritic complexity at the recent time point in the ACC.

In the ACC, group WM/RAM displayed longer apical and basal dendritic length and more spines, both recently and remotely, compared to the control groups. This group also displayed more branches and increased spine density compared to the control groups. Group WM/OP showed increases across most of the characteristics examined at the recent time point in the ACC, but only in basal dendrites. Group WM Only did not show increased dendritic complexity at the recent time point in the ACC. However, this group did show an increase in the number of apical branches at the remote time point.

In the CA1, group WM/RAM displayed more apical and basal spines, both recently and remotely, compared to the control groups. This group displayed longer dendritic length and more branches and spine density compared to control groups. Group WM/OP showed increases across most of the characteristics analyzed, but only at the remote time point. Group WM Only did not show increased dendritic complexity compared to the control group.

In examining published findings on dendritic complexity, it is important to note that there is a great deal of variability between laboratories (Scorcioni et al., 2004). For example, average apical dendritic lengths in the ACC in other published reports range from 800 to 1500 μm (Radley et al., 2004; Bock et al., 2005; Murmu et al., 2006; Zehle et al., 2007). The apical dendritic length in the present report ranged from 230 to 626 μm. This variance may result from age or strain differences, tissue processing protocols or neuron reconstruction software and techniques.

These results suggest competing hippocampal-dependent memories may result in more pronounced, or accelerated, increases in dendritic complexity in the ACC. Without hippocampal-dependent memories competing for hippocampal processing space, structural changes may be less distinct, or take a greater amount of time to come about. Interestingly, group WM/OP showed increases in dendritic complexity in both the ACC and CA1. As the type of OP task used in this study is not typically dependent upon the hippocampus (Shull and Holloway, 1985; Gallagher and Holland, 1992; Corbit and Balleine, 2000; Carvalho et al., 2001) we presume that there is no immediate competition between memory traces for the WM and OP tasks. However, structural changes indicative of memory storage did present themselves, suggesting that multiple memory processing may result in accelerated and/or more distinct structural changes compared to the processing of just a single memory.

The results presented in this study provide new information regarding structural changes associated with memory storage. Past studies have focused on spine density as the primary measure of structural changes (Restivo et al., 2009; Vetere et al., 2011). Specifically, Restivo et al. (2009) observed an increase in spine density in the CA1 recently, but not remotely, while an increase in spine density was observed remotely, but not recently, in the ACC. Spine density was also examined in the present experiment. Significantly increased apical spine density was noted in group WM/RAM:Recent compared to Control:Recent. In basal dendrites, group WM/RAM:Recent had a significantly higher spine density compared to Control:Recent, the WM Only groups and group WM/RAM:Remote. In the CA1, apical spine density was higher in group WM/RAM:Remote compared to Control:Remote, while basal spine density was higher in group WM/RAM:Recent compared to Control:Recent.

Our results are not consistent with the pattern of spine density found by others. A few important differences between Restivo et al. (2009), Vetere et al. (2011) and the present study should be pointed out. Firstly, the time point described as “recent” differed substantially between the current experiment and those of Restivo et al. (2009) and Vetere et al. (2011). In the present experiment, we defined recent as 8 days following the end of WM training. Restivo and Vetere defined recent as 24 h following conditioning. These represent two very different time points. It may be the case that our “recent” time point comes too late to see an increase in CA1 spine density recently, and a decrease remotely as has been noted before (Restivo et al., 2009; Vetere et al., 2011). Different behavioral training procedures were also used. Restivo et al. (2009) and Vetere et al. (2011) trained mice using a contextual fear conditioning procedure, while the present study trained rats using a water maze procedure (along with the RAM and OP in some groups). Differences in the type and duration of behavioral procedures may play a role as well. Our behavioral training procedure took 5 days (for each procedure) to train, whereas training on contextual fear conditioning can be done in a single day. This may result in differences in the timing of structural changes.

In the present study, structural changes were found in both the ACC and CA1 of the hippocampus. The only experimental difference between the different groups of rats was the behavioral training procedures they received. No probe trial was given prior to sacrifice. Thus, the morphological changes observed were not due to retrieval processes but likely associated with a memory storage process. Group WM/RAM was the only group to show consistent, significant differences in dendritic complexity at the recent time point compared to the control group in the ACC. This finding supports the hypothesis that taxing the demand placed upon the hippocampus, by training rats on two hippocampal-dependent tasks, results in structural changes indicative of memory storage, in the ACC. This is consistent with results from Wartman and Holahan (2013) that showed increased number of c-Fos positive cells in remotely probed groups compared to recently probed groups. In that report, the group trained on two hippocampal-dependent tasks (WM/RAM) and probed recently demonstrated increased c-Fos labeling in the ACC compared to the other recently probed groups, and similar to the level of c-Fos labeling noted in remotely probed groups, suggesting an accelerated reliance on the ACC.

The current study did not examine structural changes associated with training on the RAM or OP tasks alone. In future studies it would be of interest to examine the structural changes resulting from these tasks alone or reversed training on these tasks (i.e., RAM or OP training followed by WM). Future behavioral studies utilizing one-day training procedures are underway so that early structural changes in the ACC and CA1 can be examined. A one-day training procedure would also help to diminish delay differences between the end of training on a behavioral task and neuron analysis. In the present study, there was a difference in the delay between the end of behavioral training and neuron analysis in rats trained on one or two tasks. Training on the RAM or OP tasks entailed 5 days of training followed by a day of rest then sacrifice. Rats trained on only the WM task had 6 days of rest prior to sacrifice. This difference could influence dynamic dendritic characteristics such as spine density. This difference may become more substantial when examining recent vs. remote time points.

## Conclusion

Neuron reconstruction data showed robust increases in dendritic complexity in the ACC and CA1 at recent and remote time points in groups trained on two hippocampal-dependent tasks. These results suggest competing hippocampal-dependent memories may result in more pronounced, or accelerated, increases in dendritic complexity in the ACC. Without hippocampal-dependent memories competing for hippocampal processing space, structural changes may be less distinct, or take a longer amount of time to come about. The introduction of a multiple memory behavioral procedure presents an innovative method through which structural changes associated with memory storage can be examined. Introducing variations in the type and number of tasks used during training provides an opportunity to examine and contrast morphological differences throughout the consolidation process, shedding light on the processes underlying remote memory storage.

### Conflict of interest statement

The authors declare that the research was conducted in the absence of any commercial or financial relationships that could be construed as a potential conflict of interest.
